# 

**Published:** 1860-01

**Authors:** 


### THE
### CHICAGO MEDICAL EXAMINER.

### INDEX TO VOL. I.

Abortion, Criminal, in America .......... 168
" Trial for .......... 437
Action of Diuretic Remedies .......... 695
Academy of Medical Sciences, Chicago .......... 40, 96, 237, 287, 700
ADDRESSES—On Opening Med. Dept. Lind University, by Prof. N. S. DAVIS, M.D., &c. .......... 1
On Anatomy, by Prof. T. DEVILLE, M.D., &c., &c. .......... 110
Valedictory, before State Medical Society, by DAVID PRINCE, M.D., retiring President .......... 449
Valedictory, by Prof. T. DEVILLE .......... 705
Alcohol in Surgery .......... 171
" in Tubercular Diseases .......... 174
American Medical Association .......... 318, 392, 442
Anæsthetics, Revolution in .......... 176
Another New Medical School .......... 383
Apology .......... 383
Arsenious Acid in Apoplectic Congestion .......... 306
Bodies, Preservation of .......... 300
Biography of Claude Bernard .......... 359
Bribe, A .......... 438
BOOK NOTICES.—ASHTON, Rectum and Anus .......... 558
BECK, Medical Jurisprudence .......... 166
BLACKIE, Botany as an Ally of Medicine .......... 113
" Contributions to Medical Flora of Tennesee .......... 760
BOZEMAN, Button Suture .......... 477
BRINTON, Ulcer of the Stomach .......... 683
CHURCHILL, Midwifery .......... 601
CONDIE, " .......... 601
DEVILLE, Anatomy .......... 110
DIXON, Diseases of the Eye .......... 240
DRUITT, Modern Surgery .......... 600
ELWELL, Mal-Practice and Medical Evidence .......... 298
FOURGEAUD, History of Diphtheria .......... 358
GARRETT, Medical Electricity .......... 357, 474
GREEN, Catheterism of Air Passages .......... 358
GROSS, System of Surgery .......... 291
HAMILTON, Fractures and Dislocations .......... 240, 292
O'REILLY, Placenta and Nervous System .......... 602
PAGET, Surgical Pathology .......... 51
SAYRE, Coxalgia .......... 479
SLADE, Bronchitis as the origin of Lung Affections .......... 757
STORER, Criminal Abortion .......... 168
TODD, Clinical Lectures .......... 240
TOYNBEE, Diseases of the Ear .......... 295
WALSHE, Diseases of the Lungs .......... 561
WARREN, Life of J. C. WARREN, M.D .......... 356
WOOD, Introductory Lectures .......... 169

BOOK NOTICES.—WRIGHT, Medico-Legal Inquiry .......... 760
Report Chicago Charitable Eye and Ear Infirmary .......... 432
" Friends' Asylum for the Insane .......... 433
Transactions Connecticut State Medical Society .......... 433
" Illinois " " .......... 51, 756
" Indiana " " .......... 756
" Massachusetts " " .......... 478
" New York " " .......... 431
" Virginia " " .......... 357
Miscellaneous .......... 115
Address before Courtland Co. (N. Y.) Medical Society .......... 358
" " Philadelphia Medical Society .......... 760
American Journal Medical Sciences .......... 296
London Lancet .......... 296
Caffeine, &c. .......... 507
Can a Woman remain ignorant of her Pregnancy up to the time of her Delivery? .......... 570
Cellular Pathology, .......... 364
Cephalotripsy without Traction vs. Cesarean Operation .......... 441
Changes in Composition and Properties of Milk in the Human Female; also, Food most Proper for Infants, &c. By N. S. DAVIS, M.D .......... 577
Chest, Malformation of .......... 447
Chicago City Dispensary .......... 629
" Academy of Medical Sciences .......... 40, 96, 237, 287, 700
" Medical Journal and State Medioal Society .......... 314
" " " and Dr. E. B. WOLCOTT .......... 317
" Medical Society .......... 35, 100, 746
Chorea as an attendant upon Rheumatism .......... 720
Cold vs. Heat .......... 762
Cholera, The, in Spain .......... 762
Childhood, Disorders of .......... 372
Chloroform and Aconite in Neuralgia .......... 170
" and Cod Liver Oil in Scarlatina and Diphtheria .......... 172
" in Reduction of Dislocations .......... 304
Clinical Reports and Lectures... 55, 116, 121, 151, 155, 225, 278, 420, 423, 551, 597, 689, 747
" Researches on the Action of Diuretic Remedies .......... 695
Clinique—Rush Medical College .......... 64
Colleges, Medical, 1859-60 .......... 444
" of Pharmacy, Chicago .......... 62, 127
" Rush Medical .......... 64
" " Annual Announcement .......... 187
" Ohio Medical .......... 634
" and Charity Hospital, N. Y .......... 632
Cold Water Treatment in Laudanum Poisoning. By Prof. H. WARDNER, M.D .......... 548
Consumption .......... 380
Convention of Delegates from Medical Colleges .......... 442
Convulsions, Puerperal .......... 25, 29, 309, 725
Correspondence of Prof. W. H. BYFORD .......... 415
Coroner's Investigation in Suspected Case of Poisoning by Homœopathists .......... 736
Croup and Diphtheria. ORRIN SMITH, M.D .......... 257
Delirium Tremens, Rational Treatment of .......... 614
Diagnosis between Pregnancy and Abdominal Tumors .......... 369
Dietetic Inventiveness of Hunger .......... 439
Diphtheritis, Saline Injections in .......... 50
" Notes on, by Prof. J. H. HOLLISTER, M.D .......... 94, 148
" and Scarlatina, Chloroform and Cod Liver Oil in .......... 172
" Epidemic in California, by WM. L. WELLS, M.D .......... 193
" and Croup, by ORRIN SMITH, M.D .......... 257
" Per Chloride of Iron in .......... 305
" as it occurred on the Watershed between Tallahatchie and Miss. Rivers.... 498

Diphtheritis, Discussion on, in N. Y. Med. and Surg. Society........................ 493, 562
" " " in N. Y. Medico-Chirurgical College........................ 743
" Letter on, from GEO. W. SLACK, M.D........................ 502
Dissecting Wounds........................ 440
Disinfectant........................ 293
Diuretic Remedies, Action of........................ 695
Drowning in a Water Bed........................ 440
De Witt County Medical Society........................ 744
Elephantiasis of Clitoris........................ 252
Enchondroma of Great Toe........................ 251
Epilepsy........................ 633
" Hydrocyanate of Iron in........................ 446
Ethics, Question of........................ 188
EXAMINER Independent of all Schools........................ 124
Effectual Use of the Sponge Tent in Sterility........................ 763
Ethereal Instillations into the Ear for the Cure of Deafness........................ 763
Extirpation of Parotid Gland........................ 65, 126
Experimental Physiology........................ 125
Eye, Diseases of........................ 240
Favor, A........................ 128
Fœtal Auscultation........................ 371
Female Breasts, Inflammatory Affections of. By Prof. BYFORD........................ 513
Fractures and Dislocattons........................ 240
FISHER vs. STONE........................ 765
Flora of Illinois........................ 128
Gastrotomy........................ 308
Gelsemin........................ 307
Glycerine in Surgery........................ 303
" Iodized in Skin Diseases........................ 304
Goitre Cured by Bromide of Potassium........................ 247
Gum Elastic Air-Bag, Use of, by Prof BYFORD........................ 220
Heart's Action, Interruption of. By B. WOODWARD, M.D........................ 145
" " not Muscular........................ 297
Hernia Testis from Strumous Deposit........................ 249
Hemorrhage from the Mouth occurring Periodically. F. K. BAILEY, M.D........................ 716
" Parturient........................ 617
Hæmoptysis, Turpentine in........................ 248
" Occurring Periodically........................ 718
Hip Disease; its Treatment. Prof. E. ANDREWS, M.D........................ 751
Hospital Practice.—Pennsylvania Hospital........................ 608
Hospital, A, for Negroes........................ 763
HUNTER, JOHN........................ 764
Hydrocyanic Acid. Prof. F. MAHLA, PH. D........................ 589
Hydrocyanate of Iron in Epilepsy........................ 446
Hydrophobia Treated with Calomel........................ 177
Hypophosphites in Phthisis........................ 308
Illinois State Medical Society........................ 127, 186, 256, 314, 342, 381, 572
Illustrations of Hospital Practice........................ 608
Importance of Functions of Skin in Pathology and Treatment of Tubercular Consumption. 491
Infantile Pathology and Therapeutics........................ 608
Influence of Slow-Lead Poisoning on the Product of Conception........................ 438
Inversion of Uterus........................ 732
Iodide of Potassium in Diseases of the Brain-........................ 308, 374
Iodine Injections in Ovarian Cysts........................ 611
Lecture on Surgical Pathology........................ 51
" on Extirpation of Parotid Gland. Prof. DEVILLE........................ 65
" " " (Editorial)........................ 127
" on Erysipelas. Prof. ANDREWS........................ 151
" on Typhoid Fever. Prof. DAVIS........................ 155

Lecture on Medical Subjects........ 169
" on the Medical Profession. Prof. DAVIS........ 321
" on Parturient Hemorrhage........ 617
Legal Responsibilities of Medical Men. Dr. PRINCE........ 449
Legislation on Medical Subjects........ 528
Leptandrin as a Substitute for Mercury........ 310
Library and Museum Medical Department Lind University........ 575
Lind University, Medical Department of, Opening Address before........ 1
" " Announcement........ 63
" " Public Commencement of........ 185, 253
" " Summer Course of........ 188
Longevity........ 438
Lycopus Virginica........ 45
Man Eating Tigers........ 448
Medicines, Palatable........ 174
" of the Shetlanders........ 441
" Concentrated Organic. V. L. HURLBUT, M.D........ 276
Medicine in China........ 446
" Chicago, Academy of........ 40, 96, 237, 287, 700
Medical Department Lind University........ 767
" " University of Michigan........ 633
" Instruction........ 179
" " in Chicago........ 124
" Jurisprudence........ 166
" Men in Illinois, Notice to........ 445
" Profession of Illinois, To........ 445
" Schools in Chicago........ 573, 698
" Society, City of Galesburg........ 595
" " Scott Co........ 701
" Teachers' Association........ 385
Meningeal Apoplexy—Post-Mortem Appearances. Dr. H. LYSTER........ 727
Mercy Hospital Report........ 630
Method of Treating Stricture, M. JOBERT'S........ 762
Milk Sickness........ 177
Miscellaneous Items........ 511
Mortality in the Russian Capitals........ 441
Mutilation of Child by Homœopath. H. NANCE, M.D........ 222
Neuralgia, Pathology of. N. B. ROBINSON, M.D........ 333
" Stramonium in........ 594
New Instruments for Hip Disease, &c. Prof. E. ANDREWS, M.D........ 751
New York Academy of Medicine........ 691
New Military Hospital in Paris........ 441
Notes on Nursing. FLORENCE NIGHTINGALE........ 301
" Surgical Cases. Prof. E. ANDREWS, M.D........22, 90, 218, 338, 413, 546
Osteoid Cancer of the Femur........ 592
Ovariotomy, Successful Case of. Prof. BYFORD........ 459
Ox Gall in Disease. B. WOODWARD, M.D........ 470
Paraphlegia Cured by Electricity. Dr. CERF........ 550
Parisian Medical Intelligence........ 434
Physiology, Experimental........ 125
Placenta Prævia and Post-Partem Hemorrhage........ 723
Podophyllin as a Substitute for Mercury........ 310
Practical Medicine, Report of Committee on........ 649
Pregnancy, Stranguary as a sign of........ 472
" Quinine and Malaria in........ 175
Professions vs. Practice........ 191
Prolapsus of Funis........ 307
Puffing........ 631

Quinine in Trismus Nascentium. Prof. H. A. JOHNSON, M.D. .......... 146
" and Malaria in Pregnancy .......... 175
" its Remedial Powers .......... 375
Rasorian Doses of Tartar Emetic in Tetanus .......... 440
Relations of the Medical Profession to the Pablic .......... 437
Remarks on a Review of BRINTON'S "Ulcer of the Stomach," by Prof. T. DEVILLE, M.D. 683
Report on the Medical Uses of Veratrum Viride. A. HARD, M.D .......... 641
Request, A .......... 764
Resignation .......... 766
Retroversion of Uterus .......... 729
Salutatory .......... 61
Sanitary Science .......... 802
Secondary Syphilis, Transmission of .......... 571
Smoking and non-Smoking Students in the Polytechnic School of Paris .......... 763
Somnambulists Fined .......... 572
Special Hospitals .......... 571
Spina Bifida, Cure of, by Collodion .......... 45
Spymographer .......... 440
Sub Clavian Artery, Elevation of .......... 260
Surgical Cases in Hospital at Milan .......... 42
Syrup Iodide Potassium in Syphilis .......... 50
Tannin in Albuminous Anasarca .......... 49
Tapeworm .......... 761
Tetanus, Discussion on .......... 691
The Examiner .......... 767
Thoracico-Abdominal Fistula. Prof. H. WARDNER, M.D .......... 33
Tobacco, Coffee and Tea .......... 318
Traumatic Abscess of the Liver. Prof. H. WARDNER .......... 33
Treatment of Severe Burn of Neck by Extension .......... 252
Tumors. Prof. T. DEVILLE .......... 129
" Benign Cartilaginous. Prof. E. ANDREWS, M.D., &c .......... 270
Typhoid Fever. J. A. BRENNEMAN, M.D .......... 271
Typhus Fever, Pathology and Therapeutics of .......... 490
Uterine Polypus, Treatment of .......... 300
" Flexions, Origin of .......... 368
Uterus, The, and its Inflections .......... 367
Veratria in Acute Diseases of the Chest .......... 48
Veratrum Viride in Hemorrhage from the Mouth .......... 175
" " in Cases of Children .......... 312
" " Poisoning from. N. O. PEARSON, M.D .......... 277

### THE CHICAGO
### MEDICAL EXAMINER.

EDITED BY

N. S. DAVIS, M. D., AND E. A. STEELE, M. D.

PUBLISHED BY WM. CRAVENS & CO.

132 LAKE-ST., CHICAGO.

The EXAMINER will be issued during the first week of each month, commencing with January, 1860. Each number will contain 64 pages of reading matter, the greater part of which will be filled with such contents as will directly aid the practitioner in the daily practical duties of his profession.

To secure this object fully, we shall give, in each number, in addition to ordinary original articles, and selections on practical subjects, a faithful report of many of the more interesting cases presented at the Hospitals and College Cliniques. While aiming, however, to make the EXAMINER eminently practical, we shall not neglect either the scientific, social, or educational interests of the profession. It will not be the special organ of any one institution, society or clique. But its columns will be open for well written articles from any respectable member of the profession, on all topics legitimately within the domain of medical literature, science, and education.

Terms, [illegible]00 per annum, invariably in advance.

---

All letters and communications should be addressed to E. A. STEELE, M. D., Chicago, Ill.

---

### TO ADVERTISERS.

Advertisements from responsible parties, will be inserted at the following rates:

| | | | | |
|---|---|---|---|---|
| One Page, one year, payable quarterly, | | | | $20 00 |
| Half Page, | " | " | " | 12 00 |
| Fourth Page | " | " | " | 8 00 |
| Eighth Page, | " | " | " | 5 00 |

